# Tip in–light on: Advantages, challenges, and applications of combining AFM and Raman microscopy on biological samples

**DOI:** 10.1002/jemt.22744

**Published:** 2016-08-12

**Authors:** Batirtze Prats‐Mateu, Notburga Gierlinger

**Affiliations:** ^1^ Institute for Biophysics, Department of NanobiotechnologyUniversity of Natural Resources and Life SciencesMuthgasse 11/II 1190ViennaAustria

**Keywords:** AFM, biological materials, co‐located AFM Raman, confocal Raman microscopy, nanochemistry, nanomechanics, near‐field Raman, scanning probe microscopies, TERS

## Abstract

Scanning probe microscopies and spectroscopies, especially AFM and Confocal Raman microscopy are powerful tools to characterize biological materials. They are both non‐destructive methods and reveal mechanical and chemical properties on the micro and nano‐scale. In the last years the interest for increasing the lateral resolution of optical and spectral images has driven the development of new technologies that overcome the diffraction limit of light. The combination of AFM and Raman reaches resolutions of about 50–150 nm in near‐field Raman and 1.7–50 nm in tip enhanced Raman spectroscopy (TERS) and both give a molecular information of the sample and the topography of the scanned surface. In this review, the mentioned approaches are introduced, the main advantages and problems for application on biological samples discussed and some examples for successful experiments given. Finally the potential of colocated AFM and Raman measurements is shown on a case study of cellulose‐lignin films: the topography structures revealed by AFM can be related to a certain chemistry by the colocated Raman scan and additionally the mechanical properties be revealed by using the digital pulsed force mode. *Microsc. Res. Tech. 80:30–40, 2017*. © 2016 Wiley Periodicals, Inc.

## 1 INTRODUCTION

Complexity and hierarchy are the surnames of almost all biological materials. The specific arrangement of a few elements (mainly C, H, O, and N, sometimes combined with Ca, P, S, or Si) gives rise to numerous structures with high entanglement and specific functionalities. They are often anisotropic, different in each spatial direction, and indeed often very distinct when “zooming” in their structure. The distribution of components and their nature, that is, chemical composition and arrangement at the micro‐ and nanoscale determines their macroscopic characteristics and mechanical properties (Fratzl, 2005). Plenty of examples for complexity and hierarchy are known: the plant secondary cell wall found in woody tissues (Kerstens et al., 2001; Northcot, 1972; Roland et al., 1989), mineralized bone (Launey et al., 2010; Weiner and Wagner, 1998), conch shells (Menig et al., 2001), the sponge *Euplectella sp*. (Aizenberg et al, 2005) or life‐molecules such as proteins (Jaenicke, 1991), including silk (Römer and Scheibel, 2008) as well as RNA (Brion et al., 1997) or DNA (Bode et al., 2003). Finding a way to characterize also the lower hierarchical levels without afflicting any change in their native state can be very challenging. First of all looking at very small scales often demands special sample preparation and analysis methods and often numerous measurements are necessary to include different cells and tissues. Furthermore the fact that biological materials can be very sensitive to the surrounding conditions (such as pH, temperature, and humidity) special measurement accessories might be needed to observe their native state. A mild change in the pH or temperature can cause a protein to denature reversibly whereas harsh conditions will irreversibly affect its structure (Griebenow and Klibanov, 1996). Adhesion properties of adherent cells are also determined by the substrate they are growing on (Saravia and Toca‐Herrera, 2009) while mechanical properties of the wood cell wall are affected by moisture (Bertinetti et al., 2015).

Many of the characterization techniques in science are unidirectional in the meaning that only one or a small part of the whole descriptors that define a material/sample can be probed. Therefore often many different techniques are applied, but spatial correlation can be very time consuming and not straight away. Furthermore, divergent sample preparation requirements, the destructive nature of many wet chemistry approaches are several of the inconveniences when trying a multidisciplinary approach, which is essential nowadays in numerous disciplines (Andersen et al., 2011; Cloarec et al., 2008; Drent, 2003; Fowler et al., 2002; Rodríguez‐Vilchis et al., 2011; Tharad et al., 2015). The (colocated) combination of different nondestructive techniques, by which the same spot of the sample can be measured by two or more approaches is therefore the “new” trend (Moreno‐Flores and Toca‐Herrera, 2012). In the past years spectroscopic approaches have especially gained attention as main element for the combination with other *modus operandi*. Spectroscopy studies the interaction between light (of different frequencies) and matter, from which different properties and characteristics of the material can be derived (Harris and Bertolucci, 1989). Concretely Raman spectroscopy has a wide spectrum of applications, due to its non‐destructive nature (if correctly applied) and its suitability for combining with other methods such as scanning electron microscopy (SEM) (Cardell and Guerra, 2016; Timmermans et al., 2016), flow cytometry (Biris et al., 2009), or atomic force microscopy (AFM) (Apetri et al., 2006; Biggs et al., 2012; Zhou, 2010).

In the next paragraphs, the state of the art Raman microscopy in combination with atomic force microscopy will be described as non‐destructive approaches giving complementary data about on the one hand surface structure (topography) and other properties (e.g., adhesion, stiffness,….) and on the other hand the molecular structure (chemistry) of the sample.

## 2 CONFOCAL RAMAN MICROSCOPY (CRM)

The underlying principle of Confocal Raman microscopy is based on light scattering. Well known is the “Rayleigh scattering” explaining our perception of the blue sky by elastic (with the same energy in comparison to the excitation wavelength) scattering of the sunlight on the small air particles. Because the human eye is more sensitive to blue than violet light and the blue light has shorter wavelength than the rest of the VIS spectrum, it is scattered with more intensity (according to *I* α 1/λ^4^) (Milonni and Eberly, 2010). However, a small amount of light (10^−10^) is inelastically scattered (Raman scattering) with more (anti‐Stokes) or less energy (Stokes) due to interaction with matter. The population of molecules being in the ground energetic level is favoured (Boltzmann factor as function of temperature) and these molecules will be scattered with less energy (Stokes). The intensity of Stokes scattering is therefore much higher than the anti‐Stokes and thus this part of the spectrum is detected in conventional Raman spectroscopy (Parson, 2009). The energy difference corresponds to molecular vibrations (stretching, bending, torsion, etc.) and is responsible for the recorded shift^1^ in the spectrum, usually plotted in wavenumbers ( $\tilde{v}$[cm^−1^] = 10^7^/λ) (Griffiths, 2006). How easy a molecular vibration takes place is inherent to the polarizability ‐ “the ability to the electron cloud of a molecule to be disturbed.” Therefore nonpolar and symmetric molecules undergo a change in their electron distribution when interacting with monochromatic laser light and are indeed Raman active. The shift in the energy gives rise to a spectrum with different Raman bands that are characteristic for vibrations of specific bonding, functional groups and/or molecules. Depending on the type of vibration, the spring constant of the bond (or bond strength) and the weight of the atoms involved in the movement (as function of Hookés law),^2^ the energy for the motion and thus also the location of a specific band in the spectrum will be specific for the involved functional group and/or molecule. Stronger bonds (higher *k*) will cause the correspondent band (*e.g*., C=C compared to C—C) to appear at higher wavenumbers (more energy is lost from the incident light) whereas heavier atoms (higher µ) (O‐D, being D deuterium oxide, compared to O—H) will “vibrate” at lower wavenumbers. Stretching of atoms is less favourable than bending and thus will generate a higher energy loss: the scattered light will be placed at higher wavenumbers in the spectrum (big shift) (Smith and Dent, 2005).

A Raman spectroscopy system consists of an irradiation source (normally a coherent, collimated and polarized laser), a sample mount, a spectrometer and a detection system, usually a charge‐coupled device (CCD camera). The coupling of this system with a confocal microscope allows the generation of spatially resolved spectra (Raman imaging) and has improved the lateral resolution of the technique up to 250 nm (diffraction limited spatial resolution given by the Rayleigh criterion^3^) (Griffith, 2009; Hollricher, 2010). The z‐resolution can reach about 800 nm or poorer, but also depending on the instrumental set up (numerical aperture (NA) of the magnification lens, pinhole or fiber diameter) and the transparency or nature of the sample (i.e., refractive index) (Everall, 2004). The high spatial resolution, the possibility to measure in wet environment like water or buffer and easier sample preparation are some advantages compared to infrared microscopy, which also probes molecular vibration. In the VIS and IR range, in contrast to X‐rays, the sample does not suffer from direct damage. Different excitation wavelengths (from the ultraviolet to the near infrared) can be used for Raman microscopy. A green laser with 532 nm is common in routine measurements due to the easy use with conventional microscope objectives and accessories (e.g., glass slides and cover slips) together with high Raman scattering intensity. However, with this wavelength biological samples often yield high fluorescence and longer excitation wavelength for example, 785 nm, gives better results. Nevertheless toward the higher wavelength (infrared) the Raman intensity is decreasing with the fourth power and the spatial resolution decreases^3^ (Griffith, 2009; Hollricher, 2010).

Most modern equipment include a precise scan stage (often piezo driven) that allows nm‐wise movements with great accuracy for chemical imaging, that is, recording a Raman spectrum each 250 nm in *x* and *y* directions in a matrix‐like way. Each spectrum at each position represents a molecular fingerprint and by simply integrating over the specific band of a components characteristic vibration (spectral position), a false colour image of the distribution of that component can be generated (Dieing and Ibach, 2010; Geladi et al., 2010). However, for Raman images of biological systems it is often necessary to apply multivariate data analysis due to many overlapping bands (Felten et al., 2015; Gierlinger, 2014; Mujica Ascencio et al., 2016; Piqueras et al., 2015; Taleb et al., 2006). Stack scans (3D Raman imaging) can be also performed thanks to the use of z‐motorized tables when using a confocal microscope. The different focal planes can be resolved by using very narrow fibers as pinholes (Hollricher, 2010).

Every high‐speed imaging tool has some drawbacks. Signal to noise ratio of the single spectra is often reduced since a compromise has to be taken with integration time (measurement speed) and the laser intensity not to damage and/or burn biological samples during the scan. Furthermore, large amount of data are generated (1 spectrum each 0.3 µm) which needs computing capacity, external servers and data analysis and management software. Furthermore, “Raman imaging” with high spatial resolution (high numerical apertures) is very sensitive to the focal plane of the measured area which means that the investigated surface should be as flat as possible.

Although Raman microscopy offers chemical (together with structural and conformational) information with high resolution, it is not enough when dealing with phenomena, molecules and bonds from a few angstroms (small peptides) to few nanometres (DNA helix diameter). Only electron microscopy, scanning probe microscopies or super‐resolved fluorescence microscopy (Huang et al., 2009) achieve the resolution below the diffraction limit of light (Verma et al., 2010). Nevertheless not all of them deliver chemical information. Not to mention, the implementation of a method should be at least generally applicable to the majority of samples in native conditions and free of time consuming sample preparation steps and specific labelling or staining.

**Figure 1:**
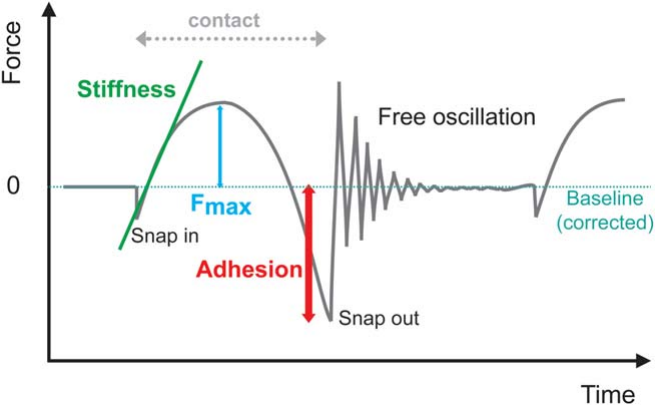
Working principle of atomic force microscopy (AFM) in digital pulsed force mode (DPFM). The cantilever oscillates free with a sinusoidal modulation at lower frequencies than its resonance frequency. At very short distances from the sample surface, the tip snaps into the sample. The repulsive forces increase as the tip pushes towards the sample and they reach a maximum (*F*_max_). From the slope of the “indentation” in the repulsive regime decreases and attractive forces originate between tip and sample, which in turn corresponds to the adhesive local forces between them. When the spring constant of the tip overcomes the adhesion forces, the tip snaps out and a new cycle begins. The baseline corresponds to long range forces and must be set to zero before any read out of absolute values.

The last condition makes scanning probe techniques more favourable. Especially Atomic Force Microscopy (AFM) (Binnig et al., 1986) has been used for decades in the visualization and mechanical characterization at the nanoscale (with about 319,000 publications since 2000). The increasing interest is due to the advantage of being a non‐damaging approach, having no tedious sample preparation requirements and providing additional information about the mechanical properties of the sample (Achterberg et al., 2014; Benitez and Toca‐herrera, 2014; Naumenko et al., 2013; Rettler et al., 2013; Strasser et al., 2007; Sundararajan and Bhushan, 2002). The AFM principle is based on Hooke's Law,^4^ which states that the restoring force of a spring is proportional to the displacement applied, or vice versa (Bhushan et al., 2004; Moreno‐Flores and Toca‐Herrera, 2012). The spring is a cantilever made of silicon (and other materials such as silicon nitride) that scans the sample's surface maintaining the force between cantilever and sample constant (contact mode) (Binnig et al., 1986). One of the most used methods works under the assumption of keeping the oscillation amplitude constant by exiting at a frequency near the resonance frequency of the cantilever. The tip stays in a non‐contact or intermittent contact regime (tapping or AC mode) (Hansma et al., 1994). Additional dynamic techniques have also been developed, including frequency modulation of the cantilever (Albrecht et al., 1991) or jumping mode (De Pablo et al., 1998; Moreno‐Herrero et al., 2000). In the relative new sub‐resonance method digital pulsed force modulation (DPFM) (Figure 1) the cantilever oscillates far below its resonance frequency with a sinusoidal modulation that allows higher repeat rates (up to 20 kHz). The tip snaps in and out of the sample homologous to what happens by a triangular modulation in force‐distance curves: after snip in, further approach causes the bending of the cantilever that depends on the mechanical properties of the sample, whereas in retraction the pull off force reflects the adhesive forces between tip and sample (Gigler and Marti, 2008; Gigler et al., 2007). The resolution achieved in AFM is determined by the sharpness and length of the tip, and its shape (pyramidal, conical, or bead‐like, e.g.), but also the radius of the sharp end of the tip (a few nanometres, typically 10 nm) and its softness ‐ spring constant (*k*). Tip materials or functionalization can also improve the resolution and give further information about electrical, thermal, magnetic or chemical properties (Ebner et al., 2007; Friedsam et al., 2004; Hapala et al., 2014; Wildling et al., 2011).

### 2.1 What AFM brings to Raman microscopy and vice versa

**Figure 2:**
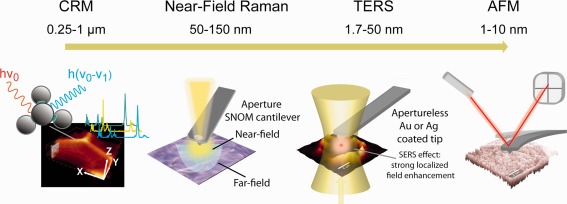
Operation principles when combining surface probe microscopies (SPM) and Raman spectroscopy. Best interpretable Chemical information is achieved applying conventional Confocal Raman microscopy, but spatial resolution is limited by the diffraction of light. Near‐Field Raman microscopy and tip enhanced Raman spectroscopy (TERS) overcome this limitation, but are more difficult to be operated on complex biological systems. Atomic force microscopy (AFM) gives structural and mechanical information on the nanoscale and is therefore in combination with Raman microscopy an important tool to reveal structure–function relationships.

AFM reveals structural (topography) and mechanical information (e.g., stiffness or Young's modulus, viscosity, local adhesion forces) on the nano‐scale complementary to the chemical information delivered by Raman microscopy on the sub‐micron scale. Therefore using both methods gives quite a complete picture and better understanding of biological systems. Both combined give additional the chance to reveal chemistry on the nano‐scale as summarized in Figure 2 and Table 1.

Integrated AFM and Raman (co‐located) was first possible in the early 2000s. Several set ups in the market are available for co‐located AFM‐Raman; some of them allow both methods to work simultaneously. While the AFM scans the top, the Raman scattering is acquired from the bottom part of the sample (bottom illumination) with a high NA objective, which limits the measurement to transparent samples only. In top illumination, another not so optimal version of the combined set up is to use special shaped tips for the AFM which enable the laser light to excite the sample from the top at the free‐tip area. However, illumination of the cantilever with near‐IR light (e.g., 785 nm) can also affect in certain degree the AFM scan (due to cantilever heating). Shadowing effect from the tip can also take place in the top illumination configuration, which affects the far field Raman signal. To overcome shadowing effects, the signal can be also collected from the side while the AFM scan is running (side‐illumination). In general, the signal when collected from the side is lower in intensity and skewed (only from one side at one specific angle) since the optimal excitation and collection angle have to be optimized (Lucas and Riedo, 2012). Top and side illumination arrangements allow only relatively low magnification (20× or 50×) objectives with high working distance (with normally NA not higher than 0.5) (Berweger and Raschke, 2010) since AFM holder and cantilever need a certain space to operate. This reduces dramatically the spatial resolution of the Raman image.

However, most of the co‐located AFM‐Raman instruments work in a one‐after‐the‐other manner. Polymer science was one of the first disciplines to benefit from the colocated measurements and a proof of principle of the methodology can be found in (Schmidt and others, 2005). After 20 years only few examples of its application in biological science/materials have come up. The “sensitive” nature of biological materials and their heterogeneity add difficulties when performing both methods at the same spot. Many biological materials need a dedicated sample preparation or fixation to be ready for successful AFM measurements and Raman imaging.

### 2.2 AFM combined with Raman in biology: From medicine to new energy generation

Combined structural and chemical data revealed conformational changes during the α‐synuclein aggregation (fibril formation) in the course of Parkinson's disease (Apetri et al., 2006). A closer look at the Raman amide I and III bands together with topographical information led to the conclusion that oligomers with α‐helical secondary structures accumulate and then undergo a conformational change to β‐sheets in protofilaments, which could be a step to control the kinetics of the fibrillization process. One of the advantages of Raman microscopy is that there are no restrictions on size or form (adsorbed, in solution) of the specimen to analyse. Extracellular polymeric substances produced in biofilms of *Acidithiobacillus ferrooxidans* grown on uranium have also been studied by micro‐Raman and AFM although no differences in the Raman spectra (composition) of the bacteria grown without uranium were found (Pradhan et al., 2008). The method is not only restricted to bacterial cells but also adherent eukaryotic cells can be imaged, for example, mechanical (changes in cytoskeleton) and chemical profiles (weaker bands) of human breast carcinoma cells at ectopic sites changed after expression of BRMS1 (metastasis suppressor), whereas the tumour site stayed intact (Wu et al., 2010). Quantification of RNA, DNA, and proteins has been done for different cells by recording Raman maps and calibration models for each component and correcting focal effects with the topography image provided by AFM (Boitor et al., 2015). Also the assessment of chemical modifications (substitutions) or mechanical processes (milling, flattening) can be studied by combining both techniques. Very often the penetration depth of certain applied modifications is not evenly distributed since it depends on diameter or the thickness of the granule or surface to be modified. This phenomenon was approached by Wetzel et al. (Wetzel, 2010) who exchanged the hydroxyl groups of waxy maize starch granules by hydrophobic octenyl‐succcinate to proportionate emulsifying functionality. The spatial distribution of the modification was monitored by Raman microscopy in combination with cluster analysis whereas AFM‐phase and ‐topography images depicted the location and functional characteristics of the modified granule. The alkaline treatment on cellulose was also studied in the same way by (Eronen et al., 2008). In medicine, this combination of methods has also been applied to inquire into drug‐drug interactions and their susceptibility to agglomerate in the carrier. Taking into account adhesive properties from force‐distance curves between propellant medium and the drug, the formulation can be optimised (Rogueda et al., 2011). Likewise the formation of a pore network during *in vitro* drug release from a carrier polymer matrix has as well been observed by co‐located AFM‐Raman (Biggs et al., 2012). Moreover catalytic reactions of metal nanoparticles have also been proofed by this technology (Harvey et al., 2012). In this case, the metal nanoparticles enhanced the Raman signal. AFM and (confocal) 3D‐Raman has been further demonstrated feasible in the vascular wall of murine aorta (Pilarczyk et al., 2014), tumour cells (Lau et al., 2014) and atherosclerotic plaque in arteries (Marzec et al., 2014). The chemical and mechanical characterization of decisive components such as elastin or lipid‐rich regions in the endothelium of the aorta and atherosclerotic plaque is possible without time‐consuming immunohistochemistry or staining. A better understanding of the tumour progress is achieved by combining the data of the volume occupied by organelles and lipid domains and the rigidity of the cells. Furthermore also research in new ways of energy generation can benefit from these methods. Anode‐grown *Geobacter sulfurreducens* were monitored during lag and exponential growth phase by AFM‐Resonance Raman spectroscopy (Lebedev et al., 2014). The measurements showed that the abundance of c‐type cytochromes is associated with the transformation of the growing pattern (lone cells to 2D and then 3D associated cells) during lag and exponential phase of their growth generating low and high current, respectively.

### 2.3 The curse of light being a wave and the way from micro‐ to nano‐Raman

As stated above, Raman microscopy resolution is limited by the wave nature of radiation (diffraction limited) (see Table 1). Light is diffracted and not focused to a point, creating a diffuse spot (Airy disk) separated by a distance given by the Rayleigh criterion (see Confocal Raman microscopy). Under this assumption, objects smaller than about half the incident wavelength of light cannot be seen (Abbe limit).

Therefore the idea of E.H. Synge was confining the photons of the incident light in a sub‐wavelength space in order to have a greater resolution. He became the father of what today is known as near‐field scanning optical microscopy (NSOM or SNOM) (Synge, 1928). SNOM is an optical scanning probe technique that takes advantage of the developed AFM technology (piezo scan tables, miniaturization of tips and beam deflection setup as feedback) and uses AFM‐like hollow probes to focus the light through a sub‐wavelength aperture and bring very close photons and sample (Figure 2). As molecules can be defined as an ensemble of dipoles, we might assume that their charge oscillates and thus they are attached to an electrical field. The whole “field” has several boundaries including a near (few nanometers from the object) and a far field (what is seen in optical microscopy). The part of the field approached in SNOM is the near‐zone due to the close vicinity between the far end of the tip and sample. The resolution of the optical image reached is then in the range from λ/10 to λ/50 nm (depending on the diameter of the aperture of the tip). The implementation of AFM probes provides also simultaneously topographical information of the sample's surface (Pohl et al., 1988).

After fulfilling the scope of getting a nanometer resolved optical image and its topographic profile, the following aim is to obtain some more information about the nature of the sample, that is, chemical composition. Near‐Field Raman Imaging is achieved by combining SNOM technology together with a very sensitive, high‐throughput spectroscopic detection system (Anderson et al., 2005). After focusing the laser light through the SNOM tip and keeping it very close to the surface, an evanescent field is formed at the end of the tip which is able to “excite” only a few nanometers on the sample (the area corresponding to the near field) (Marocchi and Cricenti, 2001). In the same manner as common Raman spectroscopy, only a very small amount of the light is inelastically scattered and the energy corresponds to characteristic vibrations of the molecules in the sample. The scattered light derives only from this small area or volume in the sample and depends on the aperture radius of the tip (Hartschuh et al., 2003). The shift in the energy of the transmitted light is collected by a high‐numerical aperture lens from the bottom.^5^ By this the Raman spectrum of very thin layers or very small sample volumes can be recorded. While the lateral resolution is highly increased, the spectral signal becomes quite low in comparison to conventional upright Raman systems.

In 2000, tip‐enhanced Raman spectroscopy (TERS) was developed as an apertureless SNOM variant (Stöckle and others, 2000). The method follows the principle of enhancing the Raman signal by approaching a metallic tip very close to the sample surface (<10 nm), similar to the effect of depositing the sample on a specially structured (rather rough) metal layer or particle like in Surface Enhance Raman Scattering (SERS) (Kerker et al., 1980; Nie and Emory, 1997). The excitation of the rough metallic surface with laser illumination originates surface plasmons that lead to strong local electromagnetic fields at very narrow positions. In TERS, the tip acts as an optical antenna that enhances the electrical field at its far end (Stadler et al., 2010; Verma et al., 2010). Tips used in TERS are metallic (normally gold or silver) and can be prepared by different methods such as vacuum evaporation, lithography or electrochemical etching (deposition of Ag on silicon cantilevers). Tip manufacturing is a limiting factor for the field enhancement, since the production is expensive and the reproducibility low. “Tip roughening” with silver structures can increase the tip radius conversely lowering the lateral resolution. Tip contamination and short lifetimes limit also the success of the TERS measurement (see also Table 1).

TERS set ups usually consist of an AFM part (on the top of the sample) and the laser excitation and signal collection system on the bottom and therefore it works best for thin transparent samples. The alignment of the tip with the laser focus can be tedious and determines together with the tip radius the lateral resolution of the measurement.

Several examples of the potential of TERS in biological samples have been demonstrated, although mainly on rather simple biological systems (Bailo and Deckert, 2008; Budich et al., 2008; Chan and Kazarian, 2011; Cowcher et al., 2016; Deckert‐Gaudig and Deckert, 2011; Hartman et al., 2016; Neugebauer et al., 2006; Schmid et al., 2013; Sharma et al., 2015; vandenAkker et al., 2015; Wood et al., 2011).

### 2.4 A case study: Colocated AFM‐Raman on cellulose‐lignin films

To not only describe biological samples, but to reveal important structure‐function relationships not only images of different structures are desired, but the mechanical properties are of utmost interest. Very few examples have focused on mechanical properties revealed by AFM and then correlated with Raman images and thus the chemical information. Here we present the potential of combining both methods in a step‐wise manner on thin films based on cellulose and lignin, which are the most common polymers on earth. Together with other polymers (hemicelluloses, pectin) they build the hierarchically structured cell walls of plants, and thus all the plant biomass. The study of their mechanical properties and their interactions with each other is important in order to understand the components that actuate in the whole tissue or plant and also for their use in new nanocomposite materials. Extracted cellulose nano‐crystals (CNC) have gained lately importance as reinforcement in material science and other fields (George and Sabapathi, 2015). Artificial lignin polymer (dehydrogenation polymer, DHP) has been also paid attention for its adhesive properties (Hoareau and others, 2006) and the enormous potential as renewable source of biomass. Films of the combined (mixed) polymers can accomplish a reductionist model about the interaction and properties of both components *in vivo*.

**Figure 3:**
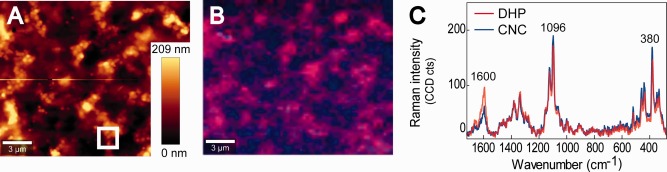
A case study of colocated confocal Raman microscopy (CRM) and atomic force microscopy (AFM). (A) AFM topography image of a film of cellulose nanocrystals (CNCs) and lignin dehydrogenation polymer (DHP) casted on a quartz window. (B) Combined false colour image based on band integration over the main aromatic stretching band at 1,600 cm^−1^ (lignin) (in red) and the main cellulose band at 380 cm^−1^ (in blue). (C) Average Raman spectra of the red (lignin agglomeration) and blue (cellulose rich) regions shown in B).

**Figure 4:**
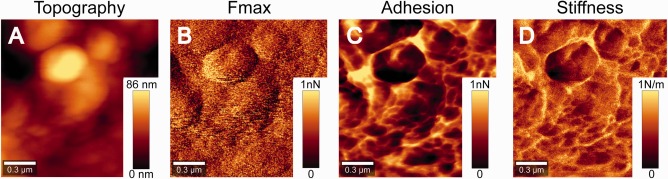
High resolution digital pulsed force mode (DPFM) on a lignin dehydrogenation polymer (DHP) agglomerate. AFM images in DPFM show (A) Topography and different mechanical properties: (B) *F*_max_ (maximum force between tip and sample), (C) Local adhesion, and (D) Stiffness. For *F*_max_, Adhesion and Stiffness, note that the values are not corrected for the tip‐sample contact area and they are plotted in relative units (minimum at 0 values, maximum at 1). The maximum force (*F*_max_) was calculated assuming that its values in DPFM (sinusoidal modulation) are homologous to the maximum force in a force‐distance curve with triangular modulation and taking into account the spring constant and the sensitivity of the cantilever.

In this case study films of casted mixed CNCs and DHPs (1800 nm thickness) have been measured on quartz substrate [details for film preparation see in (Hambardzumyan et al., 2012)] by CRM and by AFM with the Digital Pulsed Force Mode (DPFM) (Figures 3 and 4). For DPFM measurements first of all the sensitivity of the cantilever (*S* = 85.521 nm V^−1^) was measured by performing several force‐distance curves on a hard silicon surface. In the same manner, the modulation amplitude of the tip (how much from the driving amplitude is really transmitted to the tip and in last instance to the photodetector) has to be measured also on a silicon wafer before doing measurements with DPFM. AFM images were acquired on different areas (20 × 20 µm^2^ and a zoom in of 1.5 × 1.5 µm^2^ with a resolution of 256 × 256 points each. An arrow silicon cantilever with a tetrahedral shaped tip (tip radius = 10 nm) and a spring constant of *k* = 2.8 N m^−1^ was used to scan the films. The cantilever driving amplitude was optimized to 145.4 nm and the set point (maximum force) to 86.2 nN. Sampling rate was 1000 Hz and P and I gains, 3 and 6%, respectively. From the DPFM curves recorded at each point of the sample, the adhesion, stiffness and *F*_max_ images of the sample were generated. In DPFM, the local stiffness of the sample is calculated from the slope of the repulsive force signal after the tip snips in, while the adhesion image is extracted from the tip‐sample adhesive force (area under the non‐contact baseline in Figure 1) during retraction. The sensitivity of the cantilever, its stiffness and the penetration depth (ranging from 1 to 8 nm in this case) at each point of the image are required to calculate the local stiffness of the sample. Topography of the sample is generated from the feedback mechanism (*F*_max_) of the cantilever.

Raman spectra from the film were acquired using a confocal Raman microscope (alpha300RA, WITec GmbH, Germany) with a 100× objective (numerical aperture (NA) = 0.9) (Carl Zeiss, Germany). The sample was excited with a linear polarized (0°) coherent compass sapphire green laser *λ*ex =  785 nm (WITec, Germany). The scattered Raman signal was detected with an optic multifiber (100 µm diameter) directed to a spectrometer (600 g mm^−1^ grating) and finally to the CCD camera (Andor DU401A BR DD, Belfast, North Ireland). The laser power was set at 36 mW and a short integration time of 0.5 s was chosen in order to ensure fast mapping. One spectrum was taken every 0.3 μm. Images of DHP and CNCs were generated by band integration over the main lignin band at 1,600 cm^−1^ and the cellulose band at 380 cm^−1^ (CCC ring, respectively). The Control Four (WITec, Germany) acquisition software was used for both acquisitions and the ProjectPlus (WITec, Germany) software for spectral and image analysis.

Prior to the Raman measurements, AFM was performed in order to avoid possible changes due to laser overheating. In Figure 3A the Topography, based on DPFM measurement is presented of the same area given by the Raman images in Figure 3B. Topography images indicate small agglomerates on the top (from 80 to 209 nm above the bottom) of the film. The Raman images and spectra (Figure 3B–3C) reveal that these agglomerates have different chemical composition. Based on the spectra it is clear that these globules have higher amount of DHP (in red) and are clearly distinguished from CNCs richer regions (in blue). DHP shows higher intensity of the aryl C=C stretching at 1,600 cm^−1^ typical of the phenolic rings present in lignin (Agarwal and Ralph, 1997; Larsen and Barsberg, 2010). Although blue areas show this band too, the intensity is clearly decreased and cellulose bands at 1,096 cm^−1^ (antisymmetric C—O—C stretching of glycosidic bond) and 380 cm^−1^ (CCC bending in ring) (Schenzel and Fischer, 2001; Wiley and Atalla, 1987) increased. A smaller area of 1.5 × 1.5 µm^2^ comprising a DHP nodule was selected for a more detailed AFM scan (Figure 4A–4D). The diameter of the little spheres in the nodule range from 0.1 to 0.5 µm. They are connected by an interface that presents higher adhesive values (Figure 4C) and thus the more hydrophilic CNCs. The stiffness image (Figure 4D) does not resolve the nanocrystals as the scan was done on the globules revealed by Raman to agglomerate the DHP on top and a tip radius of 10nm. Yet slightly higher intensities confirm more cellulose in the bridging regions of the nodules. So the combination of AFM and Raman gives a comprehensive picture of the investigated films on surface structure, chemistry and mechanics from the submicron to the nano‐scale.

## 3 CONCLUSIONS

Raman spectroscopy has shown its feasibility for combination with other techniques over the past decades. The chemical information obtained is complementary to the mechanical/topographical images given by AFM. Their combination in TERS or near‐field Raman has high potential to monitor chemical changes at the nanoscale. However, TERS has certain drawbacks that need to be solved: tip manufacturing and reproducibility are main concerns when doing these approaches as well as sample preparation (ideally thin, homogenous in depth and transparent) and low signal Raman spectra with different bands (tip enhancement effect) that are often difficult to interpret. The fact that only a few TERS specialized groups in the world are performing successful measurements is a sign that it is not yet a straight forward method to be applied on every biological sample of interest. Colocated Raman and AFM, despite lower Raman lateral resolution than TERS, has high potential as it offers the possibility to also monitor mechanical properties of the sample and reveal structure‐function relationships. In case diffraction limited spatial resolution is not enough for chemical information, also near‐filed Raman is an option. Nevertheless again one has to struggle with less Raman signal and more restrictions on sampling.

**Table 1:** Characteristics of the different Raman‐AFM operation modes

	**CRM; (*r* = 0.61.λ/NA)**		Near‐field Raman (∼aperture size)	TERS (∼tip radius)	AFM (∼tip radius)
		SERS			
**Lateral resolution**	250 nm–1 µm		50–150 nm	1.7–50 nm	1–10 nm STM: atomic resolution
**Vertical resolution**	700 nm		1–100 nm	1–100 nm	
**Sample requirements**	–Smooth surface –No height requirements		–Flatness –Depending on the set up: transparent samples	–Smooth surface –Depending on the set up: transparent samples	–No big topographical jumps
**Best feature**: **All nondestructive**	–Depth scans, high speed –No need of labelling –Measurement in aqueous environments –Inverted set up especially suitable for cell culture measurements	–Signal enhancement up to 10^14^	–Chemistry with high lateral resolution	–Chemistry with high lateral resolution –Enhanced Raman spectra	–Many properties possible (topography, mechanics, chemistry…) –Operates in air, liquid, vacuum
**Artefacts**	–Out of focus –Burning –Change of chemistry due to laser	–Nanoparticles not homogenously distributed	–In reflexion topography affects signal intensity	–Topography effects cause different signal enhancement –Tip contamination –Sample heating can lead to irreversible changes	–Tip broadening (tip contamination) –Tip breakage –Smoothing of sharp borders
**Limitations**	–Z scans not possible in opaque samples, –Sample fluorescence	–Low reproducibility –Enhancement depends on many factors (nanoparticles, sample chemistry and surface)	–In transmission more reproducible, but thin samples necessary –Low signal	–Low reproducibility –Specimen overheating –Tip breakage –Enhanced Raman spectra difficult to interpret –Expensive tips (Au, Ag), no mass production	–Surface studies only –Tip breakage

## Footnotes

1
$\Delta \tilde{v}\;=\;\frac{1}{\lambda_{0}}-\frac{1}{\lambda_{1}}$, where Δ $\tilde{v}$ is the Raman shift measured (in wavenumbers) in the spectrum and λ_0_ and λ_1_ the wavelength of the incident and scattered photons.

2 Hookés law for a spring with attached mass: $v=\frac{1\sqrt{k/\mu }}{2\pi c}\;$ where v is the frequency of vibration, *k* is the force constant of the bond between the atoms, c is he speed of light and µ the reduced mass of the atoms involved being µ= $\frac{M1M2}{M1+M2}$

3 Rayleigh criterion: the lateral resolution is given by $r=\frac{0.61\lambda }{NA}$ where λ is the excitation wavelength of the incident laser and NA the numerical aperture of the objective through the signal is focused and collected.

4 Hookés law of a spring: *F* = −*k*. *x* where *F* is the force exerted, *k* is the spring constant and x the displacement of the spring due to the force applied.

5 All optical geometries available for SNOM and TERS are summarized and reviewed in Lucas M, Riedo E. 2012. Combining scanning probe microscopy with optical spectroscopy for applications in biology and materials science. Review of Scientific Instruments 83(6): 35.
